# Cisplatin-induced apoptosis in auditory, renal, and neuronal cells is associated with nitration and downregulation of LMO4

**DOI:** 10.1038/cddiscovery.2015.52

**Published:** 2015-11-09

**Authors:** R Rathinam, S Ghosh, WL Neumann, S Jamesdaniel

**Affiliations:** 1 Institute of Environmental Health Sciences, Wayne State University, Detroit, MI 48202, USA; 2 Department of Family Medicine and Public Health Sciences, Wayne State University, Detroit, MI 48201, USA; 3 Department of Pharmaceutical Sciences, Southern Illinois University Edwardsville, Edwardsville, IL 62026, USA

## Abstract

Cytotoxic effects of cisplatin occur primarily through apoptosis. Though several pro- and anti-apoptotic signaling molecules have been identified to play an important role in mediating the ototoxic, nephrotoxic, and neurotoxic side effects of cisplatin, the underlying mechanism is yet to be fully characterized. We reported that nitration of LIM domain-only 4 (LMO4), a transcriptional regulator, facilitates cochlear apoptosis in cisplatin-induced ototoxicity. However, its role in cisplatin-mediated nephrotoxicity and neurotoxicity is poorly understood. Therefore, HK2 and SH-SY5Y cells were used along with UBOC1 cells, to investigate the perturbations of LMO4 in cisplatin-induced cytotoxicity, in renal, neuronal, and auditory cells, respectively. Cisplatin induced an increase in the expression of active caspase-3, indicating cellular apoptosis, and increased the nitration of proteins, 24 h post treatment. Immunostaining with anti-nitrotyrosine and anti-LMO4 indicated that nitrotyrosine co-localized with LMO4 protein in cisplatin-treated cells. Immunoblotting with anti-LMO4 indicated that cisplatin induced a decrease in LMO4 protein levels. However, a corresponding decrease in LMO4 gene levels was not observed. Inhibition of protein nitration with SRI110, a peroxynitrite decomposition catalyst, attenuated cisplatin-induced downregulation of LMO4. More importantly, overexpression of LMO4 mitigated the cytotoxic effects of cisplatin in UBOC1 cells while a dose-dependent decrease in LMO4 protein strongly correlated with cell viability in UBOC1, HK2, and SH-SY5Y cells. Collectively, these findings suggested a potential role of LMO4 in facilitating the cytotoxic effects of cisplatin in auditory, renal, and neuronal cells.

## Introduction

Ototoxicity, nephrotoxicity, and neurotoxicity are among the major side effects of cisplatin, a highly effective anti-neoplastic drug used in the treatment of solid tumors.^1^ Upon entering the cell, cisplatin is converted into a highly reactive intermediate by an aquation reaction, which eventually leads to the generation of reactive oxygen species and DNA damage, resulting in apoptosis and cell death. Although these processes facilitate a reduction in tumor size and/or prevent tumor growth, they adversely affect the normal cells in the inner ear, kidney, and nervous system. Studies indicate that more than 50% of patients treated with cisplatin develop hearing loss,^2^ 70% manifest nephrotoxic effects,^3^ and 14–57% suffer from neurotoxic effects.^4^ These side effects limit the anti-cancer efficacy of cisplatin and significantly compromise the quality of life of cancer survivors. In the quest to mitigate these debilitating side effects, considerable progress has been made in delineating the signaling pathways that mediate the ototoxic, nephrotoxic, and neurotoxic effects of cisplatin.^5–10^ Though the underlying mechanisms are yet to be fully characterized, oxidative stress is widely recognized to play a causal role in the side effects of cisplatin.

Increase in nitrotyrosine or nitrite levels has been reported in cisplatin-induced ototoxicity, nephrotoxicity, and neurotoxicity.^11–13^ We identified LMO4 as the most abundant nitrated cochlear protein in cisplatin-induced ototoxicity.^5^ LMO4 is a transcriptional regulator that is involved in the regulation of cell survival and plays a major role in developmental biology. It generally functions as a scaffold protein and binds with many transcription factors to modulate their downstream signaling.^14,15^ LMO4 mediates inner ear development and is required for the normal morphogenesis of both vestibule and cochlea.^16,17^ It is also essential for development of the central nervous system, mediates calcium dependent transcription in cortical neurons, and regulates calcium release and synoptic plasticity in neurons of hippocampus.^18^ The role of LMO4 in either renal development or function is largely unknown. Our previous studies indicated that cisplatin-induced nitration of cochlear LMO4 is associated with a decrease in LMO4 protein levels^5^ and downregulation of signal transducer and activator of transcription 3,^19^ a downstream target of LMO4, and suggested that these changes facilitate ototoxicity in Wistar rats. However, the potential role of LMO4 in cisplatin-induced nephrotoxicity and neurotoxicity is yet to be clearly understood. In this study, we used three different cell lines derived from auditory, renal, and neuronal tissue, in order to determine the link between dose-dependent perturbation of LMO4 protein and the susceptibility to cisplatin toxicity.

UBOCI, HK2, and SH-SY5Y cells have been used by researchers to investigate the molecular mechanisms underlying cisplatin-induced cytotoxicity as they are susceptible to the toxic effects of cisplatin.^20–22^ UBOC1 cells are immortalized auditory sensory epithelial cells that carry a stable insertion of the conditional immortalizing gene H-2Kb-tsA58. These cells proliferate at 33 °C in the presence of *γ*IF (gamma-interferon), and differentiate when cultured at 39 °C without *γ*IF, and form regular, confluent, epithelial-like monolayers.^23^ Fully differentiated UBOC1 cells have been used as a model for cisplatin-induced ototoxicity.^20^ HK2 cells are immortalized proximal tubule epithelial cells derived from normal adult human kidney.^24^ HK2 cells have been used to delineate the signaling pathways that mediate cisplatin-induced nephrotoxicity.^25,26^ SH-SY5Y cells are derived from SK-N-SH human neuroblastoma cell line, and can be converted to many types of functional neurons.^27^ They are used as models for neurodegenerative disorders, neurotoxicity, and neuroprotection.^28,29^ Pertinent to this study, SH-SY5Y cells have been used to study the molecular mechanisms underlying cisplatin-induced neurotoxicity.^22,30^ Therefore, we used these three cell culture models to investigate the potential link between cisplatin-induced nitration of LMO4 and apoptosis in auditory, renal, and neuronal cells.

## Results

### Cisplatin induces apoptosis in auditory, renal, and neuronal cells

**Figure 1:**
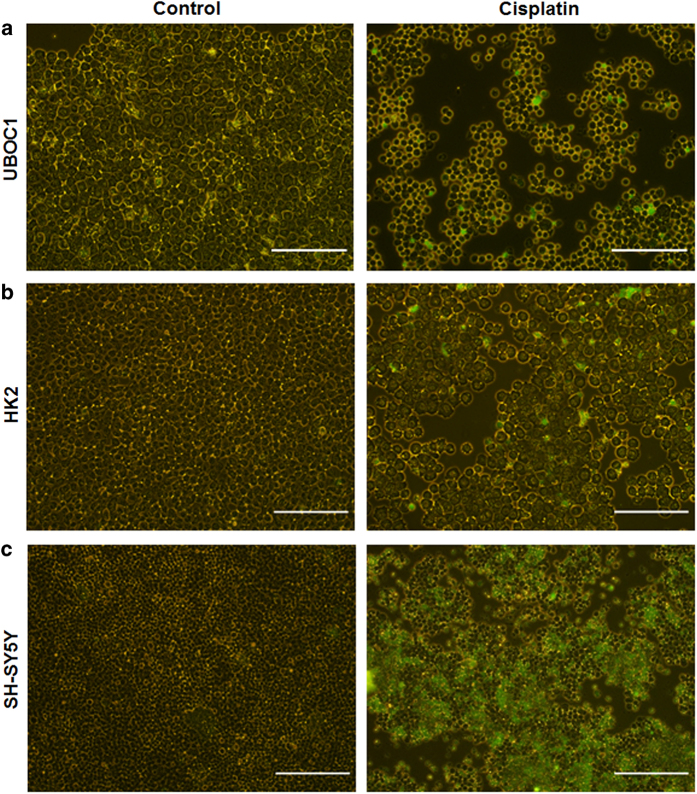
Cisplatin-induced increase in active caspase-3 expression. A fluorescein active caspase-3 stain was used to detect the expression of activated caspase-3 in (**a**) UBOC1, (**b**) HK2, and (**c**) SH-SY5Y cell cultures, 24 h post cisplatin treatment. An increase in the expression of activated caspase-3, indicated by green staining, was detected in UBOC1, HK2, and SH-SY5Y cells, after 10, 20, and 5 *μ*M cisplatin treatment, respectively. The images are representative of three biological replicates. Scale bar, 200 *μ*m.

Treatment of UBOC1, HK2, and SH-SY5Y cells with 10, 20, and 5 *μ*M of cisplatin, respectively, increased the expression of activated caspase-3, a biomarker of apoptosis, 24 h post treatment (Figure 1). Different doses were used for the auditory, renal, and neuronal cells based on the differences in their resilience to cisplatin treatment. The sensitivity of the neuronal cells to cisplatin treatment was relatively higher, while that of the renal cells was lower than the auditory cells. Nevertheless, a substantial increase in the expression of activated caspase-3, observed after treatment with the specified doses, indicated that cisplatin-induced toxicity in these cells is probably mediated by apoptosis. The specificity of active caspase-3 reaction with substrate was verified by using the inhibitor Z-VAD-FMK (Supplementary Figure 1).

### Cisplatin treatment leads to the nitration of LMO4 in cells that are susceptible to cisplatin-induced toxicity

**Figure 2:**
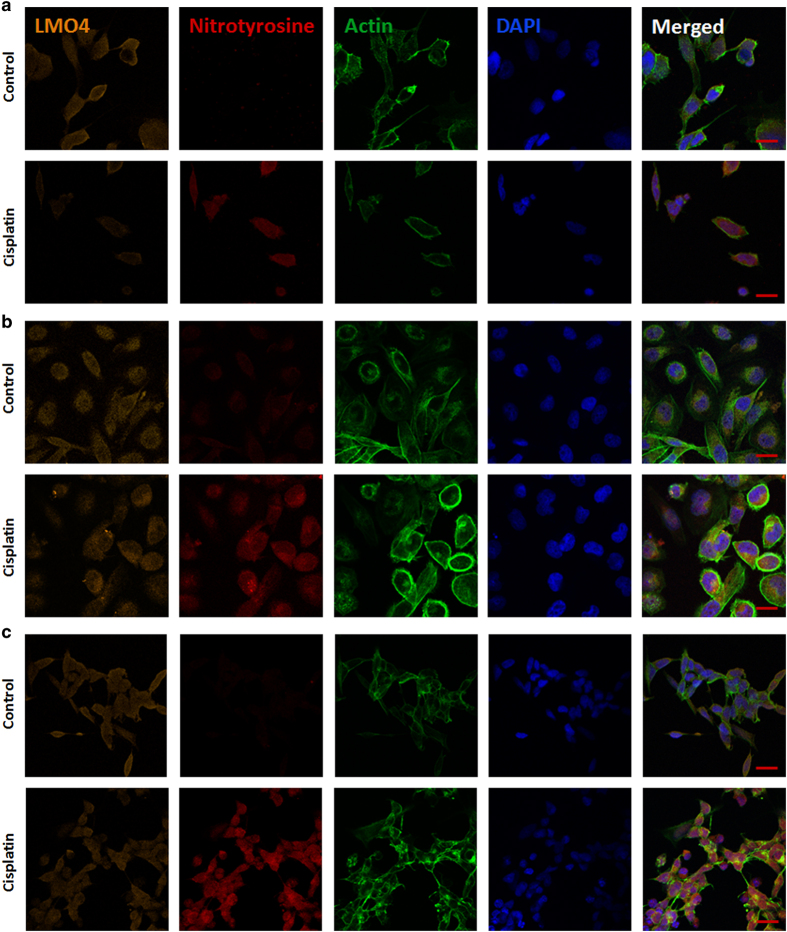
Co-localization of nitrotyrosine and LMO4 after cisplatin treatment. Co-localization of nitrotyrosine and LMO4 was assessed in (**a**) UBOC1, (**b**) HK2, and (**c**) SH-SY5Y cell cultures, 24 h post cisplatin treatment. In the panels, amber staining indicates immunoreactivity to LMO4, red indicates nitrotyrosine, green indicates actin (phalloidin), and blue indicates nuclear staining (DAPI). Treatment with 10, 20, and 5 *μ*M cisplatin induced an increase in nitrotyrosine in UBOC1, HK2, and SH-SY5Y cells, respectively. The co-localization of nitrotyrosine and LMO4 in cisplatin-treated cells was indicated by ICQ, which suggested dependent staining. The images are representative of three biological replicates. Scale bar, 20 *μ*m.

Cisplatin (10, 20, or 5 *μ*M) induced an increase in nitrotyrosine staining in UBOC1, HK2, and SH-SY5Y cells 24 h post treatment (Figure 2). Nitrotyrosine immunoreactivity was detected by immunocytochemical analysis with anti-nitrotyrosine, and the intensity of the staining was assessed using the ZEN image analysis software (Zeiss, Jena, Germany). Specificity of the immunoreaction with anti-nitrotyrosine was previously verified by the absence of nitrated protein bands after dithionite treatment, which converts nitrotyrosine to aminotyrosine.^5^ Immunocytochemical analysis with anti-LMO4 detected the expression of LMO4 protein in UBOC1, HK2, and SH-SY5Y cells. Specificity of the immunoreaction with anti-LMO4 was indicated by a faint protein band in extracts derived from LMO4 knockout HAP1 cells (Horizon genomics, #2805-12), which indicated a 86% decrease in LMO4 protein levels when compared to HAP1 controls (Supplementary Figure 2). Although several different proteins are likely to be nitrated, the intensity correlation quotient (ICQ) indicated the co-localization of LMO4 and nitrotyrosine in cisplatin-treated cells. The ICQ was +0.14±0.06, +0.27±0.07, and +0.35±0.13 for UBOC1, HK2, and SH-SY5Y cells, respectively (0<ICQ≤+0.5 indicates dependent staining).

### Cisplatin induces a dose-dependent decrease in the protein levels of LMO4

**Figure 3:**
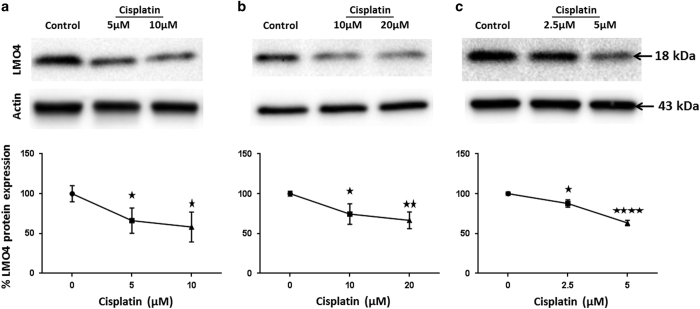
Cisplatin-induced downregulation of LMO4 protein levels. Cisplatin treatment induced a dose-dependent decrease in LMO4 protein in (**a**) UBOC1, (**b**) HK2, and (**c**) SH-SY5Y cell cultures, 24 h post cisplatin treatment. LMO4 expression was normalized with that of actin. The results are expressed as mean±S.D., *n*=3. *P<0.05, **P<0.01, and ****P<0.0001, relative to control.

Immunoblotting with anti-LMO4 indicated that cisplatin induced a dose-dependent decrease in LMO4 levels in UBOC1, HK2, and SH-SY5Y cells 24 h after cisplatin treatment (Figure 3). The cells were treated with two different doses of cisplatin. Treatment of UBOC1 cells with 5 and 10 *μ*M cisplatin induced 34 and 42% reduction in LMO4 levels, respectively, relative to controls. Similarly, treatment of HK2 cells with 10 and 20 *μ*M cisplatin induced 26 and 34% reduction in LMO4 levels, while treatment of SH-SY5Y cell with 2.5 and 5 *μ*M cisplatin induced 12 and 37% reduction in LMO4 levels, respectively. A negative correlation (*r*=−0.942, −0.956, and −0.982) was observed between cisplatin dose and LMO4 levels in UBOC1, HK2, and SH-SY5Y cells. LMO4 content was normalized to that of actin.

### Cellular levels of LMO4 mRNA are not altered immediately by cisplatin treatment

**Figure 4:**
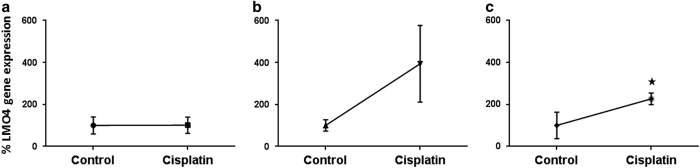
LMO4 mRNA levels were not affected by cisplatin treatment. Treatment of (**a**) UBOC1, (**b**) HK2, and (**c**) SH-SY5Y cell cultures with 10, 20, and 5 *μ*M cisplatin, respectively, did not alter the LMO4 gene levels in the auditory and renal cells, while it induced a marginal increase in the neuronal cells 24 h post treatment. Beta-actin was used as the housekeeping gene. The results are expressed as mean±S.D., *n*=3. *P<0.05, relative to control.

Reverse-transcription PCR analysis indicated that the levels of LMO4 mRNA remained unaltered or showed a marginal increase in UBOC1, HK2, and SH-SY5Y cells 24 h after treatment with 10, 20, and 5 *μ*M of cisplatin, respectively (Figure 4). The increase in LMO4 gene levels, observed in SH-SY5Y cells, occur probably to compensate for the cisplatin-induced decrease in the protein levels of LMO4.

### Inhibition of protein nitration attenuates cisplatin-induced downregulation of LMO4

**Figure 5:**
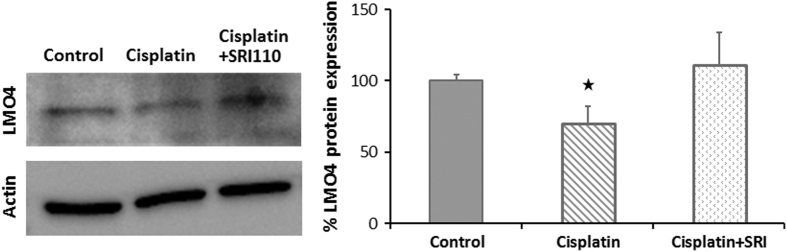
Cisplatin-induced decrease in LMO4 is attenuated by SRI110 co-treatment. Co-treatment of UBOC1 cells with SRI110, a manganese pyrrole compound, attenuated the cisplatin-induced decrease in LMO4 protein levels, 24 h post treatment. LMO4 expression was normalized with that of actin. The results are expressed as mean±S.D., *n*=3. *P<0.01, relative to control.

In order to decipher the contribution of protein nitration to cisplatin-induced decrease in LMO4 levels SRI110, a manganese(III) bis(hydroxyphenyl)dipyrromethene catalyst, was used to inhibit cisplatin-induced nitration of LMO4. SRI110 spares superoxide radical and selectively targets peroxynitrite,^31^ a precursor of protein nitration. Immunoblotting with anti-LMO4 indicated that treatment of UBOC1 cell cultures with SRI110 (50 *μ*M), 1 h before cisplatin (10 *μ*M) treatment, attenuated cisplatin-induced decrease in LMO4 expression (Figure 5). Moreover, the levels of LMO4 were not altered by treatment with SRI110 alone. LMO4 expression was normalized to that of actin. These findings suggested that cisplatin-induced nitration probably decreases LMO4 levels, while the inhibition of nitration attenuates the cisplatin-induced decrease in LMO4.

### Cell viability correlates with LMO4 protein levels in cisplatin-induced cytotoxicity

**Figure 6:**
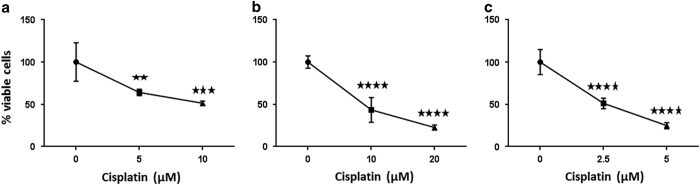
Dose-dependent decrease in cell viability after cisplatin treatment. Cisplatin treatment decreased the number of viable cells in (**a**) UBOC1, (**b**) HK2, and (**c**) SH-SY5Y cell cultures, in a dose-dependent manner, 24 h post cisplatin treatment. The results are expressed as mean±S.D., *n*=6. **P<0.01, ***P<0.001, and ****P<0.0001, relative to control.

Cisplatin-induced decrease in cell viability was assessed by using trypan blue staining. A dose-dependent decrease in the number of viable cells was detected in UBOC1, HK2, and SH-SY5Y cells 24 h after treatment with cisplatin (Figure 6). Treatment of UBOC1 cells with 5 and 10 *μ*M cisplatin induced 36 and 47% reduction in the number of viable cells, respectively, relative to controls. Similarly, treatment of HK2 cells with 10 and 20 *μ*M cisplatin induced 57 and 77% reduction in cell viability, while treatment of SH-SY5Y cells with 2.5 and 5 *μ*M cisplatin induced 49 and 75% reduction in cell viability, respectively. A negative correlation (*r*=−0.964, −0.966, and −0.985) was observed between cisplatin dose and cell viability, in UBOC1, HK2, and SH-SY5Y cells. More importantly, cell viability strongly correlated with the levels of LMO4 in UBOC1, HK2, and SH-SY5Y cells (*r*=0.997, 0.999, and 0.936, respectively), indicating a potential link between the cellular levels of LMO4 and the susceptibility of the auditory, renal, and neuronal cells to cisplatin-mediated toxicity.

### Overexpression of LMO4 mitigates cisplatin-induced cytotoxicity

**Figure 7:**
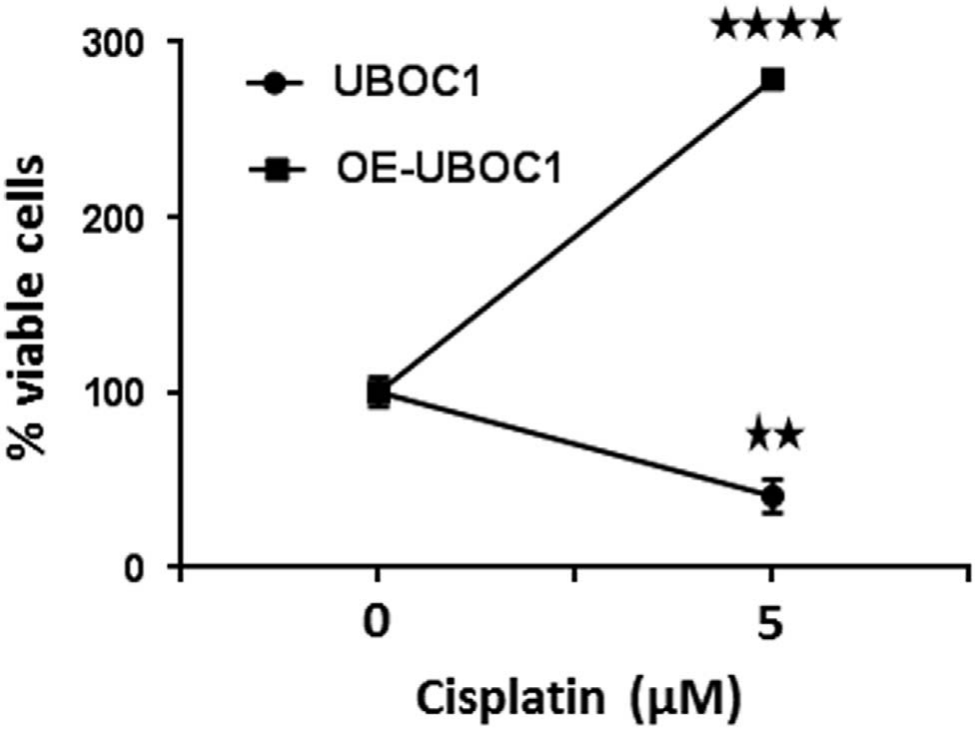
Overexpression of LMO4 prevented cisplatin-induced decrease in cell viability. Cisplatin treatment significantly decreased cell viability in UBOC1 cells. However, the number of viable cells increased significantly, 24 h post cisplatin treatment, in LMO4-overexpressed cells (OE-UBOC1). The results are expressed as mean±S.D., *n*=3. **P<0.01 and ****P<0.0001, relative to control.

To further understand the role of LMO4 in cisplatin-induced cytotoxicity, UBOC1 cells were overexpressed with LMO4, using pRK5 mammalian expression vectors, and treated with 5 *μ*M cisplatin. Transient overexpression of LMO4 not only prevented cisplatin-induced cytotoxicity at this dose but also significantly increased the number of viable cells 24 h post treatment (Figure 7), indicating a potential anti-apoptotic role of LMO4. Transfection efficiency was verified by immunoblotting the HA tag attached to exogenous LMO4 in transfected UBOC1 cells (Supplementary Figure 3).

## Discussion

Delineation of signaling pathways that regulate the cytotoxic effects of cisplatin is essential to identify interventional targets that could be manipulated to prevent its side effects in cancer survivors. This study indicated that cisplatin treatment leads to the nitration of LMO4, not only in auditory cells but also in renal and neuronal cells. A dose-dependent decrease in the protein levels of LMO4 was observed immediately after cisplatin treatment, which is in agreement with previous reports that indicate nitrated proteins are potential targets for proteolytic degradation.^32^ The mRNA levels of LMO4 were not decreased at the corresponding time point. More importantly, the cisplatin-induced downregulation of LMO4 strongly correlated with the viability of UBOC1, HK2, and SH-SY5Y, indicating a role of LMO4 in facilitating the cytotoxic side effects of cisplatin in these cells.

Cisplatin is still a drug of choice for treating solid tumors despite the discovery of a number of anti-cancer drugs in recent years. Although the remission rates are higher with cisplatin, the quality of life in cancer survivors is significantly affected by its major side effects, which are usually mediated by apoptosis. In this study, we detected a cisplatin-induced increase in the expression of active caspase-3 in UBOC1, HK2, and SH-SY5Y cells. Cisplatin-induced generation of reactive oxygen species and consequent apoptosis in sensory epithelial cells has been well documented in the inner ear.^6^ Cisplatin-induced oxidative stress, inflammation, and apoptosis have been reported to directly injure the renal tubules, renal vasculature, and glomeruli.^33^ Cisplatin is also known to damage the peripheral nerves and dorsal root ganglia neurons, facilitated by progressive accumulation of DNA adducts, inhibition of DNA repair pathways, and apoptosis.^10^ Therefore, the detection of activated caspase-3 in cisplatin-treated auditory, renal, and neuronal cells is consistent with these studies, and is in agreement with cisplatin-induced apoptosis previously reported in UBOC1, HK2, and SH-SY5Y cells.^20–22^ Moreover, the current results indicated that the dose at which apoptotic responses were detected in these cells was optimal for investigating the molecular signaling that regulates the cytotoxic side effects of cisplatin.

Nitrosative stress is emerging as a critical factor that can trigger apoptotic signaling in susceptible cells.^34^ Cisplatin-induced nitration and nitrosylation of proteins have been reported in the cochlea, and nitrated proteins were localized to cells known to be targeted by cisplatin, particularly outer hair cells.^5,35^ Cisplatin-induced increase in nitrotyrosine was detected in damaged renal tubular cells in mice, 72 h post treatment.^12^ Nitrite levels were reported to be significantly elevated in the brain of cisplatin-treated mice.^13^ Consistent with these reports, cisplatin induced a significant increase in nitrotyrosine, an indicator of oxidative damage to proteins, in UBOC1, HK2, and SH-SY5Y cells. Protein nitration can cause vital changes in biological function of the cell by modulating phosphorylation cascades, facilitating proteolytic degradation of the nitrated proteins, and altering protein function. Therefore, the significance of protein nitration in cisplatin-induced cytotoxicity depends on the functional characteristics of the nitrated protein.

In previous studies, though multiple proteins were detected to be nitrated in cisplatin-induced ototoxicity, LMO4 was identified as the most abundant nitrated cochlear protein in Wistar rats and it appeared to play a role in facilitating the ototoxic side effects of cisplatin.^5^ As a molecular adapter for protein–protein interactions, LMO4 can control gene expression by modulating the formation of transcriptional complexes. Though several reports have clarified the regulation of cellular apoptosis by LMO4,^36,37^ its role in cisplatin-induced cytotoxicity is yet to be fully understood. In this study, the ICQ analysis indicated the co-localization of nitrotyrosine and LMO4 in cisplatin-treated UBOC1, HK2, and SH-SY5Y cells. Furthermore, a dose-dependent decrease in LMO4 protein levels was observed in these auditory, renal, and neuronal cells. However, a corresponding decrease in LMO4 gene levels was not observed. Inhibition of protein nitration by SRI110 attenuated the cisplatin-induced downregulation of LMO4. Although additional studies are needed to determine whether nitration of LMO4 leads to degradation of the protein, the cisplatin-induced increase in nitration and decreased levels of LMO4 has the potential to alter LMO4 transcriptional activity, by compromising its upstream regulation and downstream signaling. Repression of LMO4 has been reported to inhibit the proliferation of epithelial cells and induce apoptosis.^37^ Consistent with this report, the dose-dependent decrease in LMO4 protein levels strongly correlated with a cisplatin-induced decrease in the viability of UBOC1, HK2, and SH-SY5Y cells. More importantly, overexpression of LMO4 in UBOC1 cells attenuated the cisplatin-induced decrease in cell count indicating a probable link between LMO4 protein levels and cell viability in cisplatin-induced cytotoxicity.

Taken together, this study suggests that cisplatin-induced nitration and downregulation of LMO4 plays a potential role in facilitating the apoptotic responses and cell death in auditory, renal, and neuronal cells that are susceptible to cisplatin-mediated toxicity. The results of this study are in agreement with our earlier reports, which indicated that cisplatin-induced nitration of cochlear LMO4-facilitated ototoxicity in Wistar rats^5^ and extended our previous findings by detecting cisplatin-induced downregulation of LMO4 not only in auditory cells but also in renal and neuronal cells. However, further proof is needed to determine whether cisplatin-induced nitration and consequent decrease in LMO4 is a common feature in all cells that are susceptible to the toxic side effects of cisplatin. If proven, targeted inhibition of LMO4 nitration and/or LMO4 degradation, in susceptible cells, could be a potential therapeutic strategy to prevent the side effects of cisplatin.

## Materials and Methods

### Reagents

All chemicals and reagents including cisplatin (P4394) were purchased from Sigma-Aldrich (St. Louis, MO, USA), unless noted otherwise. Media for cell culture, which includes minimum essential medium (MEM) with GlutaMAX (41090-036), Eagle’s MEM (30-2003), keratinocyte serum-free media (17005-042), and F12 medium (11765-054) were purchased from Life Technologies (Grand Island, NY, USA). The reagents for western blot were purchased from Bio-Rad Laboratories, Inc. (Hercules, CA, USA) and Thermo Fisher Scientific (Rockford, IL, USA). SRI110 was synthesized according to our literature report.^38^

### Cell culture

UBOC1 cells (derived from C57 BL6 mice) were provided by Dr. Mathew C Holley (University of Sheffield, UK). UBOC1 cells were initially cultured in MEM supplemented with GlutaMAX, 10% fetal calf serum, and 50 U/ml *γ*IF (315-05, PeproTech, Rocky Hill, NJ, USA) to facilitate proliferation and then cultured without *γ*IF for a week to facilitate differentiation. HK2 (CRL-2190) and SH-SY5Y cells (CRL-2266) were purchased from American Type Culture Collection (Manassas, VA, USA). HK2 cells were cultured in keratinocyte serum-free media containing bovine pituitary extract (0.05 mg/ml) and human epidermal growth factor (5 ng/ml). SH-SY5Y cells were cultured in Eagle’s MEM and F12 medium, at 1 : 1 ratio, supplemented with 10% bovine serum. HAP1 cells containing a frameshift mutation in LMO4, engineered using CRISPR/Cas9 system (Horizon genomics, #2805-12), was used as a negative control to test the specificity of anti-LMO4. The cells were cultured at 37 °C in a sterile incubator with 5% CO_2_.

### Active caspase-3 staining

CaspGLOW fluorescein active caspase-3 staining kit (88-7004) from eBioscience (San Diego, CA, USA) was used for this assay. UBOC1, HK2, and SH-SY5Y cells were plated in six-well culture plates, allowed to reach 60–70% confluence, and treated with 10 *μ*M (UBOC1), 20 *μ*M (HK2), or 5 *μ*M (SH-SY5Y) of cisplatin dissolved in phosphate-buffered saline (PBS). Twenty-four hours post treatment, the cells were re-suspended and treated with FITC-DEVD-FMK reagent following the manufacturer’s protocol. Z-VAD-FMK reagent, a caspase inhibitor, was used for the negative controls. Stained cells were visualized using a fluorescent microscope (Olympus 1X71, Tokyo, Japan) and images were acquired using DP controller software (Olympus).

### Immunocytochemistry

UBOC1, HK2, and SH-SY5Y cells were plated on two-well chamber slides (Nunc Lab-Tek II Chamber Slide system, 154461, Fisher Scientific, Pittsburgh, PA, USA) and incubated at 37 °C for 24 h. Then the cells were treated with 10, 20, or 5 *μ*M of cisplatin or PBS. Twenty-four hours post treatment, the cells were fixed with pre-cold methanol/acetone (1 : 1 (v/v)) mixture at −20 °C for 15 min. The cells were then permeabilized with 0.2% (v/v) Triton X-100 (Fisher Scientific) in PBS for 30 min, blocked (5% (v/v) goat serum, 2% (w/v) bovine serum albumin in PBS) for 1 h, and incubated with primary antibodies (anti-LMO4, sc-22833; anti-nitrotyrosine, sc-32757; both from Santa Cruz Biotechnology Inc., Santa Cruz, CA, USA) for 1 h at room temperature followed by incubation with secondary antibodies (Alexa Fluor 568 donkey anti-mouse, A10037; Alexa Fluor 647 goat anti-rabbit, A21244; both from Life Technologies) and fluorescein phalloidin (F432, Life Technologies) for 1 h at room temperature. The cells were mounted with ProLong Gold antifade reagent containing DAPI (P36935, Life Technologies), examined using the Carl Zeiss Laser Scanning Systems (Zeiss LSM 780, Jena, Germany), and images were captured and analyzed using ZEN Black imaging software (Ziess). Confocal microscopy was performed at the Microscopy, Imaging and Cytometry Resources Core at Wayne State University, School of Medicine.

### Immunoblotting

Control as well as cisplatin-treated cells were washed with ice-cold Hank’s balanced salt solution and lysed in radio-immunoprecipitation assay buffer containing protease and phosphatase inhibitors (Thermo Fisher Scientific). The protein concentration was determined by Bradford protein assay (1856209, Thermo Fisher Scientific). Thirty micrograms of protein was loaded on 4–20% Mini-Protean TGX gel (456-1093, Bio-Rad Laboratories, Inc.). The separated proteins were transferred on to a polyvinylidene difluoride membrane, blocked with 5% fat-free milk in tris-buffered saline containing 0.05% Tween 20 (Sigma-Aldrich), and incubated overnight with rabbit anti-LMO4 (1 : 1000). Then the blots were incubated with peroxidase-conjugated anti-rabbit secondary antibody (32460, Thermo Fisher Scientific), developed using chemiluminescence detection reagent (34076, Thermo Fisher Scientific), and the bands were visualized using FluorChem E System (ProteinSimple, San Jose, CA, USA). Background-corrected bands (ImageJ, NIH, Bethesda, MD, USA) were normalized against actin detected using HRP-conjugated actin antibody (sc-1615, Santa Cruz Biotechnology Inc.).

### RNA isolation and cDNA synthesis

Total RNA was isolated from control and cisplatin-treated cells using PureLink RNA mini kit (1218301A, Life Technologies). Briefly, the cells were lysed in lysis buffer, homogenized, and vortexed after adding one volume of 70% ethanol. The homogenate was transferred into a spin column, and RNA was eluted in 50 *μ*l of RNase-free water, following manufacturer’s protocol. cDNA was synthesized using a high-capacity RNA-to-cDNA kit (4387406, Life Technologies), following the manufacturer’s protocol, and the synthesized cDNA was stored at 4 °C for reverse-transcription PCR application.

### Reverse-transcription PCR

The expression of LMO4 was analyzed using the primers 5′-GGACCGCTTTCTGCTCTATG-3′ (forward) and 5′-ACGAGTTCACTCGCAGGAAT-3′ (reverse). The reaction mixture consisted of 300 nM primers, 50 ng RNA equivalents of the template, and Power SYBR master mix (4367659, Life Technologies). Real-time PCR was performed in StepOnePlus Real-Time PCR Systems (Applied Biosystems, Grand Island, NY, USA), and amplification was done in 40 cycles of 15-s denaturing at 95 °C, 60-s annealing at 60 °C. Fold changes of mRNA levels were calculated from Ct-values of LMO4 gene using beta-actin as a housekeeping gene.

### Transient overexpression of LMO4

HA-LMO4 plasmids were generated/isolated using the mammalian expression vector pRK5 (cat. no. 22964, Addgene, Cambridge, MA, USA). UBOC1 cells at 50–60% confluence were transfected with HA-tagged LMO4 using Lipofectamine reagent (Invitrogen, Carlsbad, CA, USA). Transfection of the plasmid DNA was verified by immunoblotting with HA-Tag (6E2) mouse antibody (Cell Signaling, Danvers, MA, USA). The transfected cells were cultured for 48 h and then used for the experiments.

### Cell Viability assay

Cell viability was assessed using the cell count after trypan blue staining. Twenty-four hours post treatment, control and cisplatin-treated cells were trypsinized, mixed with trypan blue stain (1 : 1), and the cells were counted on a Neubauer chamber under a light microscope.

### Data analysis

The results are presented as mean±S.D. The ICQ was calculated by subtracting 0.5 from the ratio of the number of positive PDM values to the total number of pixel values, where PDM refers to the product of differences in staining intensity from the mean intensity. GraphPad Prism 6 software (La Jolla, CA, USA) was used to employ two-tailed *t*-tests and analyze the significant difference between control and cisplatin-treated groups. The correlation between cisplatin-induced changes in LMO4 levels and cell viability was determined using the Pearson’s correlation coefficient at 95% confidence interval.

## Abbreviations

LMO4: LIM domain-only 4
*γ*IF: gamma-interferon
ICQ: intensity correlation quotient
PCR: polymerase chain reaction
MEM: minimum essential medium
PBS: phosphate-buffered saline
PDM: product of differences from mean.
