# Supplementary figures and images for: Cisplatin-induced apoptosis in auditory, renal, and neuronal cells is associated with nitration and downregulation of LMO4
Source: Cell Death Discov. 2015 Nov 9;1:15052–. doi: 10.1038/cddiscovery.2015.52 (PMC4765951; doi:10.1038/cddiscovery.2015.52)

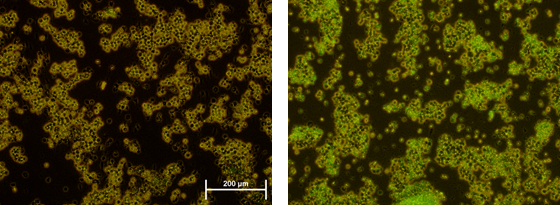

Supplement: Supplementary Figure 1 [file cddiscovery201552-s1.tiff]

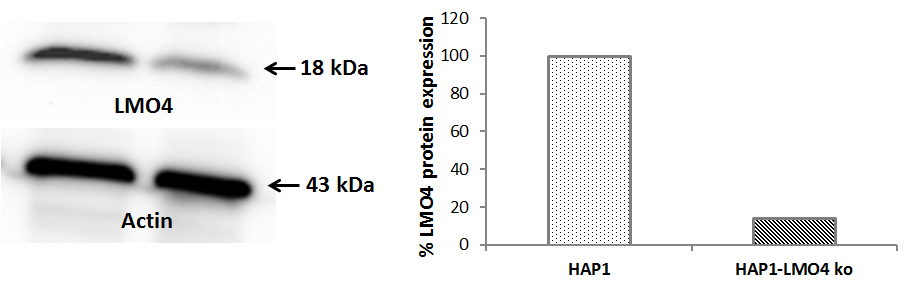

Supplement: Supplementary Figure 2 [file cddiscovery201552-s2.tiff]

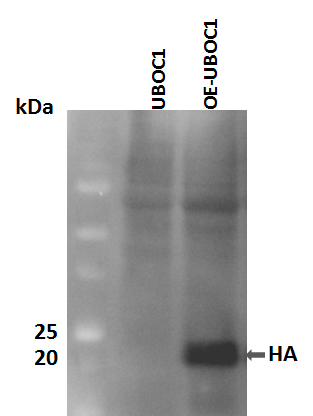

Supplement: Supplementary Figure 3 [file cddiscovery201552-s3.tiff]
