# Supplementary material for: Cisplatin-induced apoptosis in auditory, renal, and neuronal cells is associated with nitration and downregulation of LMO4
Source: Cell Death Discov. 2015 Nov 9;1:15052–. doi: 10.1038/cddiscovery.2015.52 (PMC4765951; doi:10.1038/cddiscovery.2015.52)
Supplement: Supplementary Figure Legends [file cddiscovery201552-s4.doc]

**Supplementary Figure 1. Validation of the specificity of caspase assay**

Cisplatin-treated SH-SY5Y cells were incubated with (left panel) or without (right panel) caspase inhibitor Z-VAD-FMK, prior to treatment with FITC-DEVD-FMK reagent and images were obtained using a fluorescent microscope. The absence of green fluorescent staining in the inhibitor treated cells (left panel) indicated the specificity of active caspase 3 reaction with substrate. Scale bar = 200 µm.

**Supplementary Figure 2. Validation of the specificity of LMO4 antibody using HAP1-LMO4 knock out cells**

LMO4 protein expression was quantified in HAP1 and HAP1-LMO4 knock out cells by Western blotting. The data shows a 86% decrease in LMO4 protein levels in HAP1-LMO4 knock out cells when compared to HAP1 cells, indicating the specificity of the LMO4 antibody used in this study. LMO4 expression was normalized with that of actin.

**Supplementary Figure 3. Validation of transient transfection of LMO4**

UBOC1 cells were transfected with HA-tagged LMO4 using mammalian expression vector pRK5. Immunoblot shows the expression of HA tag in transfected cells (OE-UBOC1), but not in control cells, indicating the transient transfection of LMO4.
